# Creation of a black hole bomb instability in an electromagnetic system

**DOI:** 10.1126/sciadv.adz4595

**Published:** 2025-11-05

**Authors:** M. Cromb, M.C. Braidotti, A. Vinante, D. Faccio, H. Ulbricht

**Affiliations:** ^1^ School of Physics and Astronomy, University of Southampton, SO17 1BJ, Southampton, UK.; ^2^ School of Physics & Astronomy, University of Glasgow, G12 8QQ Glasgow, UK.; ^3^ Istituto di Fotonica e Nanotecnologie - CNR and Fondazione Bruno Kessler, Povo, I-38123 Trento, Italy.

## Abstract

The amplification and generation of electromagnetic radiation by a rotating metallic or lossy cylinder, first proposed by Zel’dovich in the 1970s, is closely linked to quantum friction, energy extraction from rotating black holes, and runaway mechanisms such as black hole bombs. Although advances such as acoustic analogs of the Zel’dovich effect and the observation of negative resistance in low-frequency electromagnetic models have been reported, genuine positive signal gain, spontaneous emission of electromagnetic waves, and runaway amplification have not previously been verified. Here, we provide the first experimental demonstration that a mechanically rotating metallic cylinder acts as an amplifier of a rotating electromagnetic field mode. Moreover, when combined with a low-loss resonator, the system becomes unstable and operates as a generator seeded only by noise. The exponential runaway amplification of spontaneously generated electromagnetic modes is observed, establishing the electromagnetic analog of the Press-Teukolsky black hole bomb and paving the way to experimental tests of quantum friction from vacuum fluctuations.

## Teaser

Random fluctuations generate an electromagnetic field that is exponentially amplified.

## INTRODUCTION

In 1971, Yakov Zel’dovich predicted that an absorbing axially symmetric body rotating at rotational frequency *f* and scattering incident waves of angular momentum order *m* could somewhat counter-intuitively amplify those waves if their frequency *f* satisfies (*1*)

f<mF (1)

This condition is equivalent to the rotational Doppler-shifted corotating mode frequency ( $f_{-}=f-\mathrm{mF}$ ) becoming negative in the body’s rotating frame. While Zel’dovich considered the case of a metal cylinder scattering electromagnetic (EM) waves (*2*, *3*), he emphasized that this amplification [also known as rotational superradiance (*4*)] is a general effect rooted in thermodynamics and should therefore hold true for any rotating absorber.

His prediction was directly inspired by the Penrose process, a means of extracting energy from the ultimate absorber: a rotating black hole. The rotating spacetime creates an ergoregion around the black hole horizon, where matter and waves can have negative energy. Penrose envisioned that an object scattering within this ergoregion could split into two and lose a negative energy component into the black hole, while the positive energy part escapes, having gained energy from the black hole rotation (*5*, *6*). Black hole rotational superradiance has been tested recently in analog systems in the laboratory (*7*–*9*), and these energy extraction processes have been proposed as part of a mechanism producing relativistic jets of quasars (*10*, *11*).

This link between the Zel’dovich effect and black hole thermodynamics holds also in the quantum realm. Zel’dovich predicted that a rotating absorber could spontaneously amplify EM fields out of the quantum vacuum (*3*), ceding its rotational energy and slowing down. This implication directly inspired (*12*) Hawking’s famous prediction that even without rotation, any black hole should slowly radiate its energy away (*13*, *14*). However, this quantum vacuum rotational amplification is very weak and hence difficult to observe. For this reason, Zel’dovich speculated that forming a low-loss resonator by encircling the cylinder with a mirror could amplify this very weak signal (*1*, *2*). This generation mechanism was detailed further in 1972 with Press and Teukolsky’s “black hole bomb” concept (*11*, *15*, *16*). A rotating black hole scatters and superradiantly amplifies the impinging modes that satisfy Eq. 1. Surrounding the black hole with a mirror will reflect scattered modes back toward the hole to be reamplified. If the mirror is sufficiently reflective, then the energy lost at the mirror can be smaller than the energy gained from the black hole. With this positive feedback, these amplified signals grow exponentially, and the system becomes unstable to any random noise seed. The field energy trapped by the mirror grows until it is either released through a controlled opening (another proposed power source) or, if unchecked, until the mirror can no longer take the pressure and explodes. A third mechanism is also possible: Cardoso *et al.* (*16*) showed that the exponential amplification can also be switched off if the black hole loses too much angular momentum before the mirror explodes; at which point, the system is no longer unstable and the condition in Eq. 1 is no longer satisfied (*16*). The same instability conditions and behaviors can also occur with Zel’dovich’s EM cylinder case (*16*).

Amplification from a rotating absorber was successfully demonstrated for acoustic waves (*17*, *18*). For the case of EM waves, notwithstanding the substantially more prohibitive experimental conditions, a recent work measured Zel’dovich amplification by showing that the rotation of a metallic cylinder induces a negative resistance in an EM circuit (indicating that amplification is occurring, even if losses still dominate the overall behavior) (*19*, *20*).

In this work, we present an experimental study that relies on a rotating magnetic field generated by a three-phase stator with an internal spinning metallic cylinder. The three-phase arrangement allows us to generate a magnetic field on the internal cylinder that has a definite rotational direction. At the same time, the external circuit with the stator also acts as a reflector. Thus, the system satisfies the experimental conditions speculated by Zel’dovich for the observation of spontaneous generation and also the conditions outlined by Press *et al.* (*15*) for black hole bombs. The experimental conditions implemented show net amplification despite the internal losses of the circuitry. We also observe the spontaneous generation of waves, seeded only by background noise. This generation exhibits a runaway exponential growth, also known as self-oscillation, of the EM waves in analogy to a black hole bomb. Last, by modifying the operating conditions of the rotating cylinder, we also observe the regime, described by Cardoso *et al.* (*16*), where this instability and the exponential amplification switch off due to loss of rotational energy in the cylinder.

## RESULTS

### Experimental setup

**Fig. 1.**
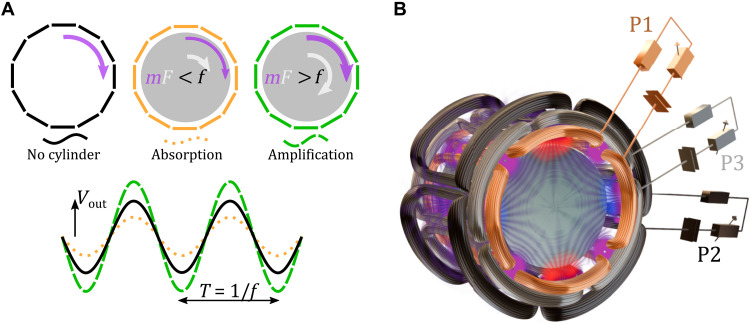
Concept and schematic layout of experiment. (**A**) Diagram showing the Zel’dovich amplification condition. For a given input field, the measured output amplitude depends on the presence of the (absorbing) metal cylinder and its rotation speed: With no cylinder, there is no effect; with the internal cylinder in place and rotating slower than the rotating EM field, the absorption is increased; if the cylinder rotates faster than the EM field, then amplification occurs. (**B**) Schematic overview of the full experiment. Three sets of external coils surround the internal aluminium cylinder. Each coil is driven by an RLC circuit (P1, P2, and P3) with a variable resistor that is used to tune the losses. We plot the numerically calculated magnetic field lines (at a given instant) in purple on the cylinder. Red and blue areas indicate north and south magnetic poles, respectively.

Figure 1A shows a visualization of the Zel’dovich amplification condition, Eq. 1. There is no effect in the absence of the cylinder; an increased absorption if the cylinder rotates slower than the angular phase velocity of the rotating field mode, f/m (purple arrow); amplification occurs only if the cylinder is rotating in the same direction and faster than f/m . These three conditions are schematically shown in Fig. 1A as three different oscillating voltage amplitudes that we measure from our circuit.

**Fig. 2.**
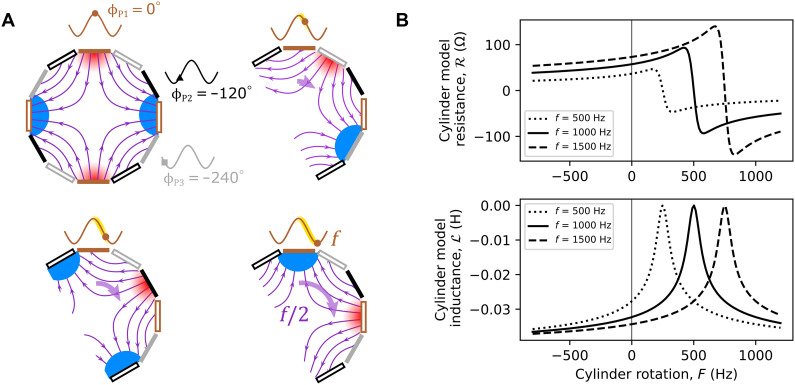
Rotating magnetic field and amplification conditions. (**A**) Numerically simulated field lines (purple) inside the coils for four different phase values of the P1 current (P2 and P3 are retarded by −120° and −240°, respectively). The North Pole (indicated as a red shaded area) at the top coil rotates by one-fourth of a cycle when the circuit currents vary by one-half a cycle [i.e., the magnetic field spatially rotates at half the frequency ( f/2 ) of the current *f*]. The cylinder rotation only needs to outpace, e.g., a North Pole of the field to meet the amplification condition. (**B**) Theory: The graphs show the resistance and inductance of the internal cylinder only as a function of the rotation frequency of the internal metal cylinder for three different external quadrupole EM field rotation frequencies. A corotating cylinder will lead to a negative cylinder resistance (hence amplification) above the threshold F=f/2 and increased resistance (hence increased losses) below this threshold.

Figure 1B shows a schematic overview of the experimental setup. A rotating magnetic field is generated by a three phase induction motor, consisting in a stator with three independent resistance-inductance-capacitance (RLC) circuits (P1, P2, and P3). An aluminium cylinder, spun by a brushless DC motor, is nested inside the stator, with only a small ~1-mm air gap separating it from the stator (for details on the experimental apparatus, see Methods). The magnetic field is rotated in time by ensuring that the three circuits are 120° out of phase with respect to each other, and the relative sign of the phase shifts determine the direction in which the magnetic field rotates. The design of the coils determines the shape of the magnetic field lines and leads to a quadrupole rotating mode. This field is numerically simulated and shown as an overlay in purple on the cylinder for a fixed time instant in Fig. 1B. The quadrupole rotating mode implies that we have a mode with orbital momentum *m* = 2, i.e., the field has a phase factor $∼{\text{exp}}^{i\left(m\varphi -\omega t\right)}$ with *m* = 2. Thus, the spatial pattern of the field completes half a rotation for every 2π cycle of the sinusoidal current in the circuits, as illustrated in more detail for four different times across a half-cycle of the current in Fig. 2A.

In our experiment, each RLC circuit is also used to store EM energy at its resonant frequency, *f*_res_. This resonance acts as a lossy mirror (analogue to the black hole bomb mirror) confining the EM radiation around the cylinder, thus increasing the interaction.

Figure 2B shows how the effective resistance and inductance of the cylinder in the rotating quadrupole mode (*m* = 2) is predicted by our model to change with cylinder rotation rate, *F* (model details are reported in Methods). When the cylinder is corotating with the mode (*F* > 0), we see a transition to negative resistance (i.e., loss transitions to gain) when F>f/2 (we show curves for three different values of *f*). For F=f/2 , the cylinder rotates at the same frequency of the field *B*, so it sees a stationary field. Consistently with this picture, the model predicts that the resistance induced by the cylinder will vanish. The model also predicts that the inductance changes with rotation, and this changes the RLC resonant frequency accordingly. We note here that a negative resistance in the cylinder does not imply a net positive amplitude gain in the full system (e.g., in the circuits P1, P2, and P3), as these will always have an additional resistance and hence a loss term that needs to be overcome by the cylinder amplification. We therefore purposely include a variable resistor *R*_var_ in each circuit with which we can tune the RLC losses.

An important detail in the following is the operation regime of the motor driving the internal metallic cylinder. We choose two different measurement settings for the motor drive to observe two distinctly different regimes and better understand the energy exchange dynamics between the mechanically driven internal cylinder and the EM field in the stator: (i) a closed-loop setting, where we fix the motor rotation frequency *F*—this setting will allow us to observe net Zel’dovich amplification and reach the unstable black hole bomb regime; (ii) an open-loop setting, which allows the motor rotation rate to change—in this case, we will observe wave generation and exponential amplification from noise and then the switching off of the unstable regime due to loss of rotational energy in the cylinder.

### Zel’dovich amplification and black hole instability threshold

**Fig. 3.**
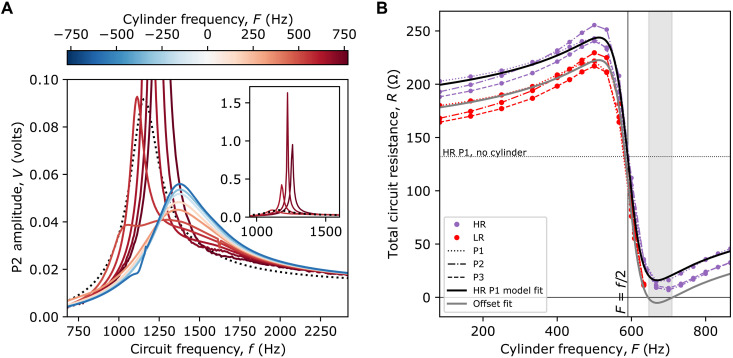
Experimental results: Closed-loop motor settings. (**A**) Shows measured RMS voltage (*V*_o_) for increasing EM wave frequency and for varying cylinder rotation rates (blue curves indicate counter-rotating and red curves indicate corotating). Also included is no cylinder case (black dotted line), which peaks at 0.0916 V. The colors show the directionality of the effect—for opposing directions of cylinder rotation (positive and negative F), the effect on the rotating mode in the circuit is different. Only the corotating cylinder (red) is able to amplify above the no cylinder peak, see Eq. 1. The inset shows the highest amplitude peaks, which are an order of magnitude above the no cylinder case. A peak gain factor of 17.6× is observed for the case of the cylinder rotating at 700 Hz. (**B**) Total resistance *R* in the three circuits P1, P2, and P3 for a fixed circuit frequency *f* = 1181 Hz and different cylinder rotation frequencies *F*. Purple dots are data for high resistance circuits, and red dots for low resistance circuits. The vertical line indicates the Zel’dovich threshold rotation, and the dotted horizontal line indicates the $R_{\text{circ}}=131\;\;\Omega$ measured P1 resistance without cylinder (for the high resistance case). The numerical model fits to P1 high resistance data is also plotted (black solid line), with the coupling strength *A* and the constant resistance present without cylinder *R*_circ_ as free fit parameters. We find $R_{\text{circ}}=129\;\;\Omega$ , which matches the measured value well, and *A* = 0.397. The gray line is the fit offset by ΔR=−21.2Ω for P1 ( $\Delta R=R_{\text{var}}^{\text{LR}}-R_{\text{var}}^{\text{HR}}$ ), indicating the expected total resistance in the low resistance case. The shaded region indicates the range of cylinder frequencies where we expect to see a net exponential amplification of a *f* = 1181-Hz signal.

We first set the cylinder motor at a fixed rotation speed in the closed-loop configuration. We choose a relatively high value for the variable resistor *R_var_* ≈ 24 ohms (Table 1 in Methods) so as to ensure that overall, the total loss (circuit plus cylinder) dominates over the cylinder gain for all conditions. Figure 3A shows the voltage amplitude in circuit P2 as we vary the EM frequency *f* in the circuit (similar results are obtained in all three circuits). The various curves are for different rotation frequencies *F* of the cylinder, as indicated in the legend. A clear resonance is observed in the “no cylinder” case (black dotted curve)—this resonance arises, as discussed, from the RLC circuit and is determined by the choice of inductance and capacitance values. It is around this resonance that we have energy accumulation in the circuit, akin to energy accumulation in a cavity that enhances the interaction of the EM field with the cylinder. It is around this resonance that we focus our attention. We see variations in the resonant peak amplitude as a result of the cylinder rotation and different behaviour depending on the direction of the cylinder with respect to the rotating mode. The blue curves in Fig. 3A indicate the counter-rotating cases, which all exhibit lower maximum amplitudes compared to the no cylinder case (dotted curve), i.e., the presence of a counter-rotating cylinder causes loss. The red curves indicate the corotating cases and lead to reduced losses with very significant loss-reduction factors, i.e., >10× when Eq. 1 F>f/2 is satisfied, i.e., for F≳600 Hz (see three highest peaks in Fig. 3A inset). Furthermore, the resonant peak frequency also shifts with the cylinder rotation speed: The resonance frequency is minimum for ∣f/2−F∣=0 , when the cylinder is corotating with the field or there is no cylinder (dotted curve) and increases for ∣f/2−F∣>0 due to the additional cylinder inductance, as also discussed in Fig. 2B.

**Table 1.** Input and component values for all three circuits. *R*_0_ is given at 20°C, but the coils heat up during the experiment, increasing the resistance (details in the Supplementary Materials). The temperature coefficient of resistance α for the copper wires is ≈0.004/°C.

Circuit	*C* (nF)	*V*_i_ (mV, RMS)	*R*_0_ at 20°C (ohms)	$R_{\text{var}}^{\text{HR}}$ (ohms)	$R_{\text{var}}^{\text{LR}}$ (ohms)	Phase (rad)
P1	149.9	12.69	71.6	22.4	1.2	0.013
P2	149.7	12.70	71.4	27	1.0	−2.0915
P3	149.7	12.67	71.4	23.7	1.1	2.0975

Figure 3B shows the measured total circuit resistance for all three P1, P2, and P3 circuits for varying cylinder rotation rates at a fixed circuit EM frequency of 1181 Hz. The solid black curve indicates the numerical model fit (Eq. 18 in Methods) with *R*_circ_ = 129 ohms (*R*_circ_ is the circuit resistance without the cylinder). The model fits the data well and the positive resistance values confirm that overall, the total system (cylinder plus circuit) is not amplifying. We note that the total resistance approaches zero around *F* = 680 Hz, indicating a nearly perfect cancellation of the circuit positive resistance with the cylinder negative resistance. By decreasing the value of the variable resistors *R*_var_, we expect these curves to shift downward by the change in *R*_var_, ∆*R*. In this way, we can push the curve around *F* = 680 Hz into a region of total negative resistance, i.e., reaching absolute gain. These measurements are also shown for all three circuits (red curves), overlaid with the ∆*R*-shifted numerical model (gray curve). As can be seen, the measurements do not actually continue into the negative resistance region: Any attempts to perform the same measurements at cylinder rotations *F* > 640 Hz led to a runaway amplification causing the circuit resistor to explode, a very eloquent hint that the black hole bomb regime has taken over.

The runaway behavior and explosions are a direct result of driving the motor with fixed rotation speed in the closed-loop configuration, i.e., when the rotational energy is extracted from the cylinder, the feedback loop feeds new power into the motor to keep it at the same speed. Thus, the system can continuously draw increasing amounts of power from the cylinder motor to feed the increasing voltage and current in the circuits.

### Noise amplification and self-limitation of the instability

**Fig. 4.**
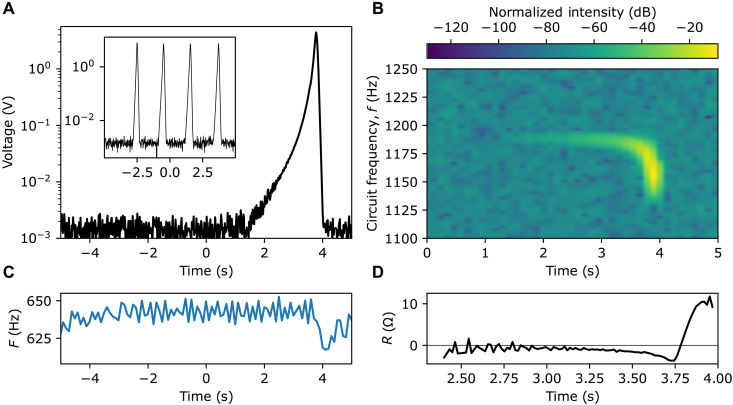
Experimental results: Open-loop configuration. (**A**) Increase of the voltage measured over the 5-ohm resistors in the circuits, on a log scale, when the cylinder is set to rotate at *F* = +643 Hz and driven in the open-loop feedback configuration. No input signal is supplied to the circuits. We show the envelope of the signal measured in time, bandpass filtered to the 1100- to 1250-Hz band shown in (B). The inset shows the circuit evolution over an equal measurement time for the cylinder driven to *F* = +660 Hz, which grows more rapidly, evidencing the cyclical self-halting and amplifying behavior of the system. (**B**) Spectrogram of the measured signal (decibel taken with reference to max value), highlighting that the signal is seeded by the noise floor in a narrow band of frequencies that correspond to the most negative total resistance. (**C**) Measured cylinder rotation rate *F* over time, exhibiting a marked decrease in correspondence to the exponential growth of the circuit voltage; this is an evidence of the system converting mechanical rotational energy into EM energy. (**D**) Total resistance *R* extracted from the signal that is negative (in the initial linear region around −0.75 ohms) until the cylinder slows down sufficiently that the cylinder gain (negative resistance) is overcome by the circuit resistance, resulting in a positive total net resistance.

The exponential trend of the instability can be measured by changing the measurement settings to the open-loop configuration while keeping *R*_var_ at the low value to have a net total gain. The circuit can now only feed off the rotational energy of the cylinder for a limited time before slowing it down, and the cylinder rotation rate will then drop below the instability condition and the total resistance will return to positive values, switching off the instability before the resistor explodes. Furthermore, we remove the circuit input signal, leaving only noise as a seed. The measurements in this regime are shown in Fig. 4. We set the cylinder to rotate at 643 Hz such that we are just inside the negative frequency region for our circuit (shaded area in Fig. 3B). Figure 4A shows the time trace of the voltage in circuit P2: Initially, we observe only the noise floor while the cylinder is rotating at a constant speed (see Fig. 4C). A growing signal, initially masked by the detection noise floor, appears after a few seconds as a result of amplification of the circuit noise. We measured the phase of the spontaneously generated field and find that it co-rotates with the cylinder, as predicted for a signal generated through this black hole bomb instability (see the Supplementary Materials). The inset in Fig. 4A shows the circuit dynamics for a higher cylinder rotation (660 Hz) and so greater number of times there is an increase by a factor \it{e}, where we see the oscillation between stable and unstable dynamics due to the motor periodically slowing down as it loses mechanical energy to the circuit and then speeds up again as it falls below the exponential amplification frequency range. This regime represents the Cardoso *et al.* (*16*) prediction for black hole bomb instability for the Zel’dovich cylinder case.

**Fig. 5.**
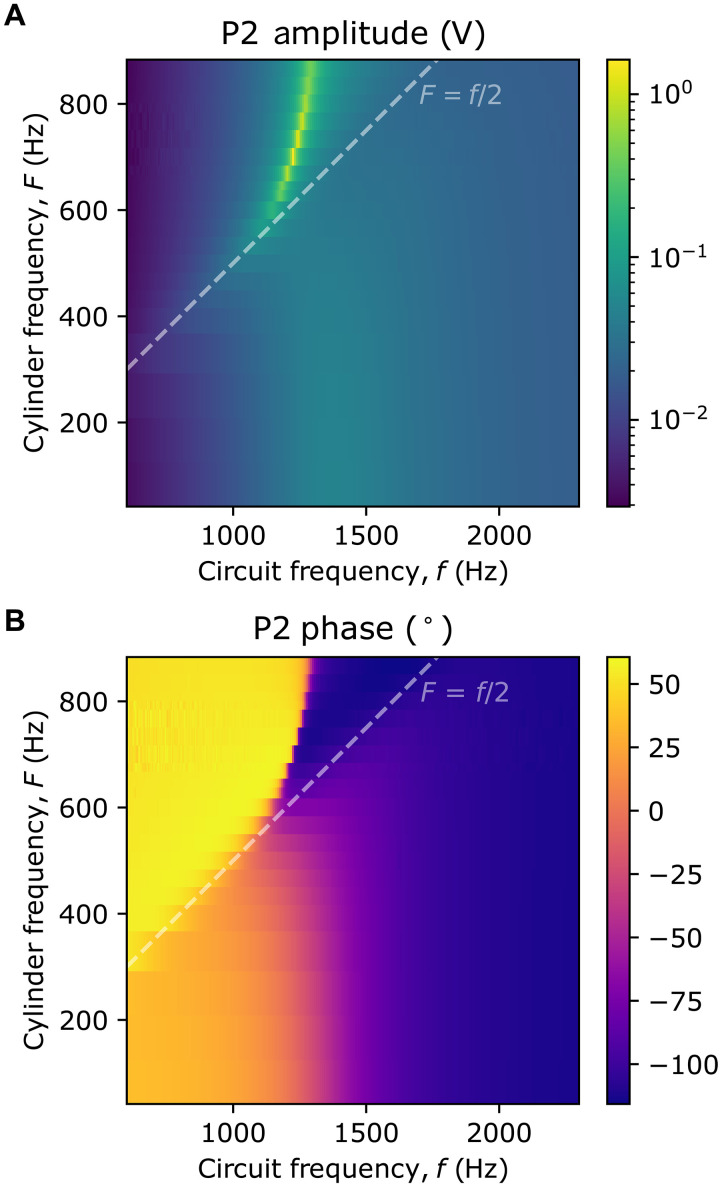
Experimental result: Circuit voltage amplitude and phase for varying cylinder frequency as well as cylinder and circuit frequency. The high resistance P2 amplitude (**A**) and phase (**B**) dataset for different frequencies and rotation speeds. The Zel’dovich amplification threshold F=f/2 is indicated with a dashed line. The resonance peak changes in both frequency and amplitude with rotation speed.

We also see in Fig. 4B that the amplified signal shifts to lower frequency values before the amplification switches off. A decrease in cylinder rotation speed, observable in Fig. 4C, causes the resonance of the circuit to shift toward lower frequencies (see Fig. 5 and Methods). This process continues until the cylinder speed becomes too low and exits the instability condition (the total negative resistance region), and the signal dissipates away. Another interesting feature in Fig. 4A is the observation of an initial exponential growth rate that at later times becomes superexponential. This superexponential behavior corresponds to a nonlinear decrease of the total resistance *R* in Fig. 4D and signals a clear departure from the standard black hole bomb theory (*1*, *2*, *15*, *16*). These nonlinearities of the system will be investigated in future research. Apart from the nonlinearities at high amplitude, the amount of total negative resistance in Fig. 4D of a few ohms (using Eq. 9 in Methods) is consistent with the prediction of the numerical model shown in Fig. 3B.

## DISCUSSION

The black hole laser, predicted by Jacobson *et al.* (*21*) as a way to amplify analog Hawking radiation, and Press and Teukolsky’s black hole bomb (*15*) are examples of black hole instabilities that are enhanced by the presence of a surrounding mirror that increases the mode density in the vicinity of the unstable region (*11*). The experiments presented here are a direct realization of the rotating absorber amplifier first proposed by Zel’dovich in 1971 and later developed by Press and Teukolsky into the concept of black hole bomb. In both of these cases, the amplifier is seeded by the quantum vacuum, and this amplification of quantum fluctuations from a rotating absorber still remains to be observed experimentally. In this work, the amplifier is operated at room temperature and is therefore seeded by noise that dominates vacuum fluctuations by many orders of magnitude. Nevertheless, the physical ingredients are as proposed more than 50 years ago. The results show that extraction of rotational energy from an absorber with exponential amplification of EM waves can be observed at low frequencies, where the conditions for negative energies (or negative resistances) can be met. Furthermore, it also shows how this unstable regime can be switched on and off as predicted for the black hole bomb (*16*). A challenge for the future remains the observation of spontaneous EM wave generation and runaway amplification seeded from the vacuum. However, based on the results presented here, this now remains a purely technological (even if very hard) feat. As pointed out by Unruh, any quantum noise amplifier is “completely characterized by the attributes of the system regarded as a classical amplifier and arises out of those classical amplification factors and the commutation relations of quantum mechanics.” (*22*). A first necessary step, as shown in this work, therefore is the realization of said classical amplifiers; this work provides one possible technical solution and a route toward future experiments aimed at measuring vacuum amplification from rotation and related quantum friction effects.

## METHODS

### Experimental details

The setup is depicted in Fig. 1B. Our Zel’dovich cylinder is a 40-mm-diameter solid aluminium conductive cylinder attached to a DC motor [Maxon ECXSP19L 2 pole brushless DC motor, 0- to 900-Hz rotation rate, as in our previous work (*19*)], which provides a controllable rotation speed. This rotating absorber is surrounded by the stator of a three-phase quadrupole induction motor (Panasonic M8MX25G4YGA). The air gap between the stator and cylinder is ≈1 mm. The stator consists of three sets of four coils. Each set of four are wound in alternate directions between adjacent coils to create a quadrupole magnetic field when a current flows. If the sets are provided the same alternating current with a phase difference of 120°, then the total quadrupole field rotates. As visualized in Fig. 2A, the field rotates at half the frequency of the AC, as when the current has progressed by 180° the field has only rotated by a quarter circle (90°). The rotating quadrupole field has the topological charge *m* = 2. A Hall probe was used to confirm the rotation direction for a given phase ordering of the coil sets.

Each set of coils was connected in series with a capacitor and resistors to form a resonant RLC circuit for each phase. The combination of a capacitor (which stores energy in an electric field) and inductive coils (storing energy in a magnetic field) allows energy to oscillate between them with a resonant frequency of

$$\omega_{\text{res}}=2\pi f_{\text{res}}=\frac{1}{\sqrt{\text{LC}}}$$ (2)

While in the main paper frequencies *f* and *F* are used, in the methods section, the angular frequencies ω = 2π*f* and Ω = 2π*F* will be used for simpler expressions in the mathematical theory.

**Fig. 6.**
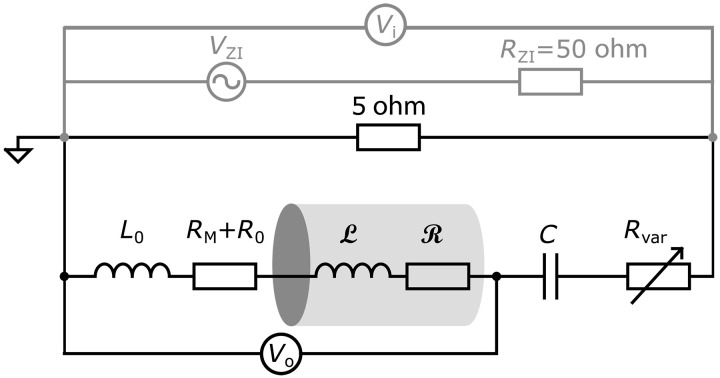
Circuit for each phase. When the ZI is connected as input signal, $R_{i}=$ $\frac{\left(50\times 5\right)}{50+5}\Omega =4.54\;\Omega$ ; otherwise, *R_i_* = 5 ohms.

The total resistance in the circuit was controlled by a fixed 5-ohm resistor and a variable resistor *R*_var_. Each phase circuit input voltage was provided by one output of a Zurich Instruments (ZI) lock-in amplifier (HF2LI), which has its own internal resistance of ≈50 ohms. Connecting the ZI output across the 5-ohm resistor, it acts as a voltage divider producing an effective source input voltage *V*_i_ and lowered effective source impedance *R*_i_ = 4.54 ohms (Fig. 6). The three-gang variable resistor was used to vary the total resistance in the circuits simultaneously. This controlled whether the system has low enough resistance for the cylinder rotation to take the circuit into a total negative resistance instability regime, without changing other elements in the circuits.

### Circuit model

The effective RLC circuit for each phase as described in the previous section is represented in Fig. 6 and includes the stator coils (*L*_0_, *R*_0_, and *R*_M_), the field interacting with the cylinder (R,L ) in series with a capacitor (*C*), a fixed 5-ohm resistor and a variable resistor *R*_var_. Values for each phase are given in Table 1.

The circuit can be modeled in a simple way by a transfer function for complex output voltage *V*_o_ (measured over the coils) from complex input voltage *V*_i_ (applied over the 5-ohm resistor)

$$V_{o}=\frac{Z_{\text{coil}+\text{cyl}}}{R_{i}+R_{\text{var}}+Z_{C}+Z_{\text{coil}+\text{cyl}}}V_{i}$$ (3)

The capacitor impedance $Z_{C}=1/\left(i\omega C\right)$ and the impedance of the coils and their interaction with the cylinder $Z_{\text{coil}+\text{cyl}}=Z_{\text{cc}}=$$R_{\text{cc}}+i\omega L_{\text{cc}}$ , where

$$R_{\text{cc}}=R_{0}+R_{M}\left(\omega \right)+R\left(\omega ,\Omega \right)$$ (4)

$$L_{\text{cc}}=L_{0}\left(\omega \right)+L\left(\omega ,\Omega \right)$$ (5)

Here, *R*_0_ is the ohmic resistance of the stator coil, *R*_M_ accounts for any other effective resistance present without the cylinder, such as mutual resistance between circuits or from induced currents in surroundings. *L*_0_ is the inductance of the coil without the cylinder, and R  and L  are the effective resistance and inductance contributed by the presence of the cylinder and its rotation, which is where the Zel’dovich effect appears.

Rearranging Eq. 3, *Z*_cc_ can be extracted from our measurements of output voltage and known circuit values

$$Z_{\text{cc}}=\frac{\left(Z_{C}\;+\;R_{\text{circ}}\right)V_{o}}{V_{i}-V_{o}}$$ (6)

The total resistance and inductance in the circuit can be found as

$$R=ℜ\left[Z_{\text{cc}}\right]+R_{i}+R_{\text{var}}$$ (7)

$$L=L_{\text{cc}}=ℑ\left[Z_{\text{cc}}/\omega \right]$$ (8)

(*R* shown in Fig. 3B) and *L* shown in the Supplementary Materials). When the cylinder is not present ( R,L=0 ), the total resistance of the circuit by itself is $R_{\text{circ}}=R_{0}+R_{M}\left(\omega \right)+R_{i}+R_{\text{var}}$ , and the inductance of the circuit itself is *L*_0_. By comparing the data taken with and without the cylinder present, the extra contributions of the rotating cylinder ( $R=R-R_{\text{circ}}$ and $L=L-L_{0}$ ) are evident.

When the circuits are exponentially amplifying, there is no measurable input signal provided, it is initially seeded by random noise, and the frequency response Eq. 3 is no longer useful. Instead, we use the time domain expression of the voltage amplitude envelope function over time *t*, which for a standard RLC circuit is given by

$$V\propto e^{-\frac{\mathrm{Rt}}{2L}}$$ (9)

### Zel’dovich cylinder model

First, from the point of view of the cylinder, a varying magnetic field impinging on a conductor induces eddy current loops in the conductor, which causes a responsive magnetization of the cylinder (a reflected field) and dissipation of the field energy. This response of a material to an applied magnetic field is described by its complex magnetic susceptibility $\chi =\chi^{\prime }-i{\chi^{\prime }}^{\prime }$ . In our case of a conductive aluminium cylinder, the dependence of the susceptibility on field frequency ω_ can be understood through the physics of penetration depth, δ

$$\delta =\frac{1}{\sqrt{{\sigma \mu \omega }_{-}}}$$ (10)

where σ is the electrical conductivity of aluminium (3.77 × 10^7^ at 20°C), μ = μ_0_μ_r_, where μ_0_ is the vacuum permeability and μ_r_ = 1.000022 for the relative permeability of aluminium. The penetration depth scales with frequency as $1/\sqrt{\omega }$ . An EM wavelength much smaller than the conducting cylinder dimensions cannot penetrate very far, and interaction with the entire cylinder is restricted to a thin layer of eddy currents on the surface; at this extreme, the reflection is strong and the absorption is weak. An EM wavelength that is very large compared to the cylinder can pass through the entire cylinder without much interaction, so both responses are weak at this extreme—same as the cylinder that is not present at all. When the wavelength is on the same order as the dimensions of the cylinder, the field penetrates far into the conductive cylinder without simply passing through, maximizing the absorption response.

When the cylinder is rotating at Ω, an external field of frequency ω with angular momentum *m* (with respect to the rotation axis) appears to have a rotationally Doppler-shifted frequency

$$\omega_{-}=\omega -m\Omega$$ (11)

in the rotating frame. One can imagine from Fig. 2A how a point on the cylinder surface, rotating around the centre, will experience a different field frequency due to the way it rotates and sweeps through the spatially and temporally varying field. The cylinder’s response function to a field with angular momentum changes correspondingly with rotation thanks to that shifted frequency changing the effective penetration depth δ(ω_).

If the cylinder is corotating fast enough, ω_ can become negative. What happens to the response in that case? Zel’dovich argues (*1*–*3*) that to avoid breaking the second law of thermodynamics when conserving energy and angular momentum, when ω_ becomes negative, the absorption (proportional to ${\chi^{\prime }}^{\prime }$ ) also must flip sign, turning into amplification. This results in an odd-symmetric absorption response about ω_ = 0.

Looking at the amplification from the point of view of the RLC circuit, the eddy currents induced in the conductive cylinder will couple a magnetic flux Φ^refl^ into the circuit. We can write the reflected flux as Φ^refl^ = α(ω_)*I*, where *I* is the current applied to the circuits, and the complex flux response function is α = α′ – *i*α″. The response function α has to be evaluated at the field frequency ω_ seen by the cylinder in the rotating frame. However, the magnetic flux reflected into the circuit will oscillate in the laboratory frame at the applied frequency ω, so the associated voltage induced in the circuit is

$$V=i\omega \Phi^{\text{refl}}=i\omega \alpha \left(\omega_{-}\right)I$$ (12)

From the complex flux response function α, we can define the resistance R  and the inductance L  generated by the presence of the cylinder as

$$R=ℜ\left[V/I\right]=\omega {\alpha^{\prime }}^{\prime }\left(\omega_{-}\right)$$ (13)

$$L=ℜ\left[\Phi /I\right]=\alpha^{\prime }\left(\omega_{-}\right)$$ (14)

To find analytical expression of α we note that from the definitions of circuit inductance *L* and of α

$$\frac{\alpha }{L}=\frac{\Phi^{\text{refl}}}{\Phi^{\text{appl}}}≃\frac{B_{r}^{\text{refl}}}{B_{r}^{\text{appl}}}$$ (15)

Here, Φ^appl^ = *LI* is the self-magnetic flux applied by the current *I*. The fields $B_{r}^{\text{refl}}$ and $B_{r}^{\text{appl}}$ are the radial components of the reflected and applied fields evaluated at the coil surface. We assume that the coils are shaped to be parallel (and very close) to the cylinder external surface and neglect border effects from the finite length of the cylinder, so the fields can be assumed to be uniform across the coil area.

Now, the problem is how to calculate $B_{r}^{\text{refl}}/B_{r}^{\text{appl}}$ , which requires solving the Maxwell equations for the cylinder with proper boundary conditions in the frame rotating with the cylinder. We use an analytical infinite cylinder model, generalizing the result derived in (*19*, *23*) for a dipolar field (*m* = 1) to fields with arbitrary angular momentum *m*. A rotating field with cylindrical symmetry of order *m* features radial and azimuthal components rotating at ω_ with an angular dependence exp(−imφ) . In empty space, the solutions are of the form $f\left(r\right)e^{-\mathrm{im}\varphi }e^{-i\omega_{-}t}$ with radial functions $f\left(r\right)\propto r^{m-1}$ or $f\left(r\right)\propto r^{-m-1}$ . The first solution can be interpreted as the field applied by the coils **B**^appl^ (vanishing at *r* = 0 for *m* > 1), the second solution as the field reflected by the cylinder **B**^refl^ (vanishing at infinity). Inside the metallic cylinder, the Maxwell equations take the form of the Bullard equations, and the radial part can be expressed in terms of Bessel functions (*23*). Using these solutions and applying the boundary conditions at the cylinder surface, after defining

$$S=\frac{B_{r}^{\text{refl}}}{B_{r}^{\text{appl}}}$$ (16)

we find

$$\begin{array}{c} S={\left(\frac{a}{r}\right)}^{2m}\frac{\left(\mu_{r}\;+\;1\right)J_{m}\left(\sqrt{i}\frac{a}{\delta }\right)\;-\;\sqrt{\frac{i}{m^{2}}}\frac{a}{\delta }J_{m-1}\left(\sqrt{i}\frac{a}{\delta }\right)}{\left(\mu_{r}\;-\;1\right)J_{m}\left(\sqrt{i}\frac{a}{\delta }\right)\;+\;\sqrt{\frac{i}{m^{2}}}\frac{a}{\delta }J_{m-1}\left(\sqrt{i}\frac{a}{\delta }\right)} \end{array}$$ (17)

Here, *J*_m_ are Bessel functions of first kind and δ is the effective penetration depth in the rotating frame $\delta =1/\sqrt{\sigma \mu \left(\omega -m\Omega \right)}$ . Note that in the latter equation *r* is the radius at which the coils are located, while *a* is the radius of the cylinder. In our setup, *m* = 2, *a* = 0.020 m, and *r* = 0.021 m, so ${\left(a/r\right)}^{2m}\approx 0.8$.

By plugging *S* in Eq. 15, we find α, and by means of Eq. 13, we find

$$R=\omega {\alpha^{\prime }}^{\prime }\left(\omega_{-}\right)=A\;\omega L\;ℑ\left[S\right]$$ (18)

$$L=\alpha^{\prime }\left(\omega_{-}\right)=A\;L\;ℜ\left[S\right]$$ (19)

where *A* is a constant of the order of 1 that can be used to take into account inaccuracies and border effects. We expect *A* < 1 since border effects imply a loss of efficiency. We have used Eq. 18 for the theoretical curves in Fig. 2B and the fitting curves in Fig. 3. In the latter case, we find an efficiency factor *A* = 0.397.

When the cylinder corotates at the same speed of the rotating field, ω_ = 0, the reflected field vanishes and *S* = 0. When the cylinder corotates faster than the rotating magnetic field, ω_ becomes negative, flipping the sign of ℑ[S] , thus changing the effective resistance of the cylinder R from positive to negative. This counteracts the positive resistance of the coil and the rest of the circuit and, if the effect is sufficiently strong, can cause the total resistance of the whole system to become negative, allowing for an exponential amplification instability.

### Experimental procedure: Stable regime

For the stable regime, the ZI HF2LI was used to supply the input signal (*V*_i_ at *f*) and measure the RMS (root mean square) output signal (*V*_o_, at the input frequency *f*) amplitude and phase with respect to the input reference. As the HF2LI models only have two outputs and two inputs each, two were used, and one locked into the other to always provide the three excitation voltages *V*_i_ at the same frequency with a fixed 120° phase difference between them. Only the HF2LI providing the initial oscillator reference could perform lock-in measurements of *V*_o_ at a reasonable speed, so while all three phases were always energised with *V*_i_ while taking data, only two phases *V*_o_ were measured at once. To get data from all phases, runs were repeated, swapping out one of the measured phases.

The dependency of the steady-state voltage output on both circuit frequency (*f*) and cylinder rotation frequency (*F*) was investigated. The cylinder frequency was set and strictly maintained with closed-loop control by the controller for the maxon DC motor. The direction of cylinder rotation could also be chosen. For a set cylinder rotation speed (in the range 0 to 900 Hz) and also for the case of no cylinder present (to determine *R*_circ_ and *L*_0_), voltage measurements were taken, while the circuit frequency was swept, usually recording at 250 points from 600 to 2600 Hz. Figure 5 shows the measured *V*_o_ amplitude and phase for the three circuits as a function of *f* and *F* when *R*_var_ has its higher value. These data were then analyzed using Eqs. 6 and 7 to extract *R* and *L* measurements.

#### Attenuated measurements

When the amplification was high, the resonance peaks would saturate the ZI’s 1-V (RMS) input range, which could lead to an underestimate of the output voltage. To avoid this, data at some rotation frequencies (667 to 800 Hz) were taken with the measurement probes on 10× attenuation mode. However, it came with the issue that the recorded voltage was not an exact 0.1 multiple of the 1× recorded voltage, but the amplitude and phase depended on magnitude and frequency. Small corrections to the conversion were needed to incorporate these few data points with the normal 1× measurements dataset.

Data taken at the same cylinder rotation (667 and 800 Hz) in both 1× and 10× modes were used to fit modified conversion functions for our amplitude and phase data

$$|V|=\left(v_{1}\;+\;v_{2}f\right){|V_{10X}|}^{v_{3}}$$ (20)

$$\phi =\phi_{10X}+p_{1}+p_{2}f+p_{3}f^{2}$$ (21)

with values for each measurement channel shown in Table 2. The noise level for those measurements is also amplified, which becomes noticeable in the derived values at the tails of the resonance peaks where the amplitude is very low (fig. S5 for example).

**Table 2.** Values for attenuated measurement conversion corrections.

Circuit	*v*_1_	*v*_2_ (s)	*v*_3_	*p*_1_(°)	*p*_2_ (°s)	*p*_3_ (°s^2^)
P1	10.82	−4.039 × 10^–4^	0.9922	0.5954	−5.53 × 10^–3^	1.507 × 10^–6^
P2	11.23	−5.069 × 10^–4^	0.9955	−4.186	0	0
P3	11.42	−5.837 × 10^–4^	0.9971	3.788	−0.01007	2.494 × 10^–6^

### Experimental procedure: Unstable regime

To investigate the unstable exponential amplification regime, no input signal was required, so the input and outputs of the ZI were removed, and the three phases were measured simultaneously with an oscilloscope (Tektronix, DPO2024B). When this voltage was measured over the coils, the oscilloscope would saturate (fig. S8), whereas measuring over the 5-ohm resistor allowed the peak voltages at the turning point to be recorded fully (Fig. 4). The cylinder speed would be set by the DC motor control to be in the exponential amplification region. Here, an open loop control (*24*) was used to set a demand speed the system would try to achieve by sending a voltage proportional to the speed and the drawn motor current. This is a slower and less strict adjustment than the closed loop control, which uses the motor’s actual speed for feedback. The open-loop control allowed the motor speed to drop below the instability threshold when the cylinder was transferring large amounts of energy to the RLC circuit, which cut off the process, this limiting mechanism saving the RLC circuits from getting fried repeatedly under the more strict maintenance of the closed-loop control.

From the oscilloscope data, the spectrogram is generated directly from the data, with the size of the FFT block chosen to produce a spectrogram with reasonable trade off between time and frequency resolution. For other measurements, a Hilbert transform is used to extract the instantaneous amplitude, phase, and frequency of the signal. This extracts the peak voltage amplitude, not the RMS ( $V_{\text{peak}}=V_{\text{rms}}\sqrt{2}$ ). An optional butterworth bandpass filter can be used to filter away the noise outside the frequency region of the signal. The data are smoothed by averaging over time (e.g., from 12.5-kHz sample rate to 62.5 Hz). The regions of each channel, where it measures within its measurement range, are combined to make a single voltage envelope signal from the three channels. *L* is calculated from the frequency using Eq. 2 and then combined with the instantaneous amplitude exponent value to calculate *R* from Eq. 9.
