# Supplementary material for: Creation of a black hole bomb instability in an electromagnetic system
Source: Sci Adv. 2025 Nov 5;11(45):eadz4595. doi: 10.1126/sciadv.adz4595 (PMC12588278; doi:10.1126/sciadv.adz4595)
Supplement: Supplementary file 1 — Figs. S1 to S10 [file sciadv.adz4595_sm.pdf]

Science Advances
AAAS

Supplementary Materials for

Creation of a black hole bomb instability in an electromagnetic system

M. Cromb *et al.*

Corresponding author: H. Ulbricht, h.ulbricht@soton.ac.uk

Sci. Adv. **11**, eadz4595 (2025)
DOI: 10.1126/sciadv.adz4595

This PDF file includes:

Figs. S1 to S10

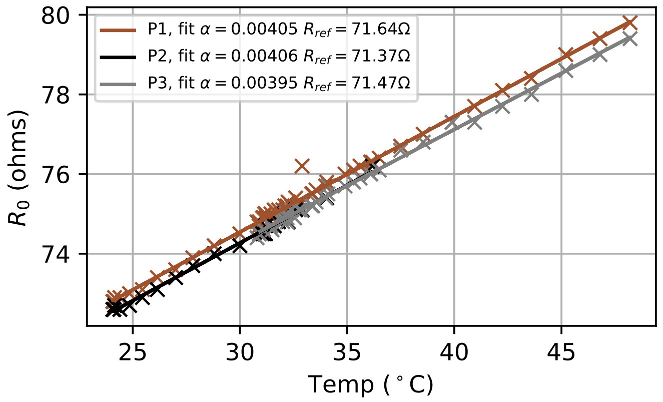

fig. S1. **Stability over multiple experimental runs:** Coil casing temperature and coil resistance data taken over several different measurement runs. A fit to Eq. S2 for $T_{ref} = 20°C$ is given.

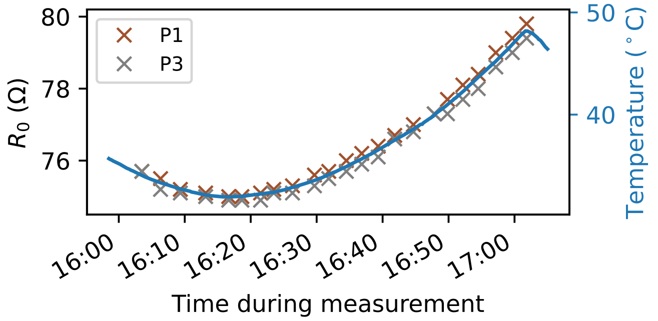

fig. S2. **Stability over experimental run:** Coil resistance and stator temperature over time during a long measurement run, during which the cylinder speed is stepped up from 0 Hz to 867 Hz.

Temperature effect on circuit

The coils would heat up when the cylinder was rotating at high speeds, this did change their resistance accordingly.

$$dR = \alpha dT R_{ref} \tag{S1}$$

$$R = R_{ref}(1 + \alpha(T - T_{ref})) \tag{S2}$$

The standard temperature coefficient of resistance α for copper at T_{ref} =20°C is around 0.004/°C. The temperature of the stator casing was measured by a thermocouple probe and the ohmic resistance of the coils taken with a multimeter during measurements, these values agreed with the standard alpha value (fig. S1).

The temperature change over a dataset would depend on how long the dataset took, i.e. the number of cylinder speeds sampled. During the longest datasets, the high resistance and positive direction runs in which the cylinder speed was increased in steps between 0 Hz and 900 Hz over the duration of around 50 minutes, the temperature of the stator casing could increase by 10-20°C. The coils were not usually left to cool to room temperature between runs, so the temperature would also cool during the slower cylinder rotation speeds of the runs (fig. S2). Even on the longest runs, the resistance did not usually vary by more than 5 ohms.

As this change in resistance is small compared to the change generated by the cylinder rotation rate, for simplicity it has not been considered in the main data analysis, where the offset resistance R_{circ} is taken to be constant over the cylinder rotation F. A temperature change would also change the conductivity σ of the cylinder, again this effect is small enough to be neglected, and the conductivity is considered constant in the Zel'dovich model. In the exponential amplification regime, the measurements are taking place over the order of seconds, so the temperature can be considered constant over the measurement.

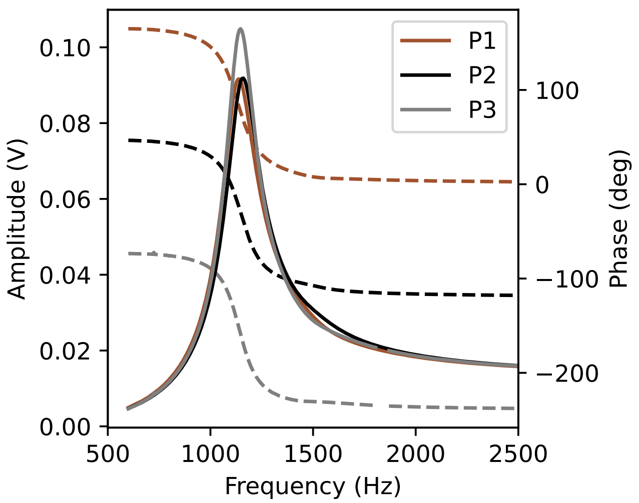

fig. S3. **No cylinder measurement data from all three phases - high resistance case.** Shows the output r.m.s. voltage V_o amplitude (solid line) and phase (dashed line) resonance over the circuit frequency range.

Inter-circuit interactions

The circuit model (Eq. 3 in the main text) generally considers each phase independently, but incorporates the inter-circuit interactions from the shared magnetic field in a limited way, through the $R_M(\omega)$ and $L_0(\omega)$ parameters for each phase, which make up part of Z_{cc}.

The need for the frequency dependence of these parameters is apparent when Z_{cc} is measured without the cylinder present ($\mathcal{R}$, $\mathcal{L} = 0$), see fig. S4. We see features for each phase centred near $f = 1500$ Hz. When the contributions of all three phases are summed together, the features cancel out into a simpler, approximately linear frequency dependence over the measured range, indicating an origin from some shared interactions between the circuits, likely due to the fact they are not exact copies.

A full treatment of the system would require a matrix approach rather than a circuit-by-circuit approach, to fully take into account the inter-circuit interaction. Nevertheless, the simple model we have used is more than adequate to reveal the presence of the Zel'dovich effect in this experiment. Furthermore, the majority of this contribution is circuit frequency dependent rather than cylinder rotation speed dependent, and so when slicing the dataset to analyse results at a constant circuit frequency (Fig. 3 in the main text) it can be approximated simply as a background offset for each phase, absorbed into R_{circ} and L_0.

Estimate of instability region

Fig. 3b in the main paper shows the measured total circuit resistance at $f = 1181$ Hz for all three circuits. The offset fit line (in grey) and corresponding shaded region indicates a range of F where, when R_{var} is low, all the circuits are expected to have a total negative resistance to a $f = 1181$ Hz signal. However, as this is only for one value of f, the range of F where the system is unstable to any f will be greater. Fig. S5 shows how the total resistance varies with both f and F in the R_{var} high case. Note that while at some f, F, some circuits individually have a total negative resistance, in this parameter region there is always one circuit (P2) that is still positive in this R_{var} high regime, and thus the overall coupled system is still stable, and is able to be measured without any exponential increase in the voltage (or things blowing up) over time. The bottom right plot in fig. S5 shows (akin to the grey offset in Fig. 3b in the main text) an expectation of where, in the low resistance case, the system would be unstable - where all three circuits are expected to have a total negative resistance. This prediction is made from offsetting the high resistance data by ΔR for each circuit, and then plotting the maximum value out of the three circuits (note this may not always be P2, due to the circuit interactions mentioned in the previous section, shown in fig. S4). The region where there is a negative resistance predicted follows the peak amplitude region e.g. in Fig. **??** in the main text.

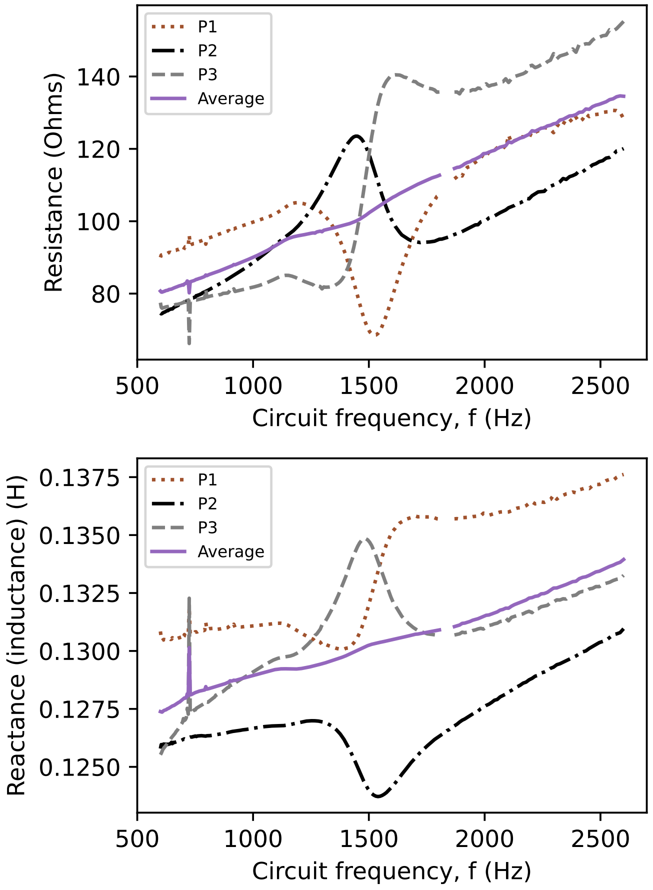

fig. S4. **No cylinder data - high resistance case.** Shows resistance R_0+R_M and inductance L_0 extracted from measurements over coils.

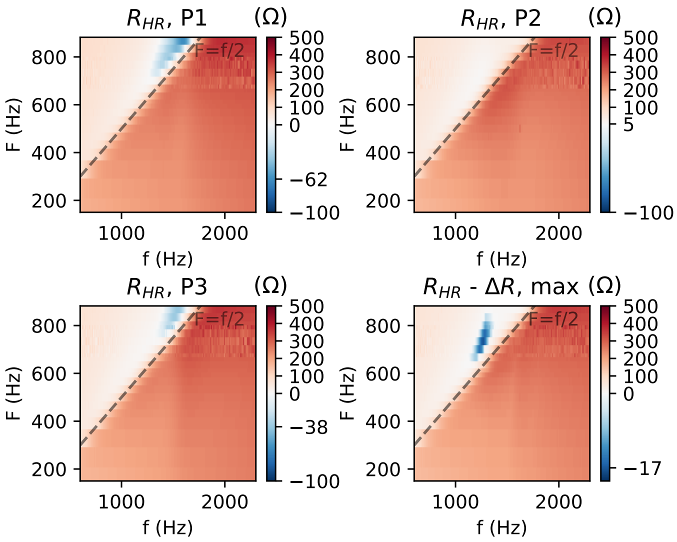

fig. S5. **High resistance datasets,** with the Zel'dovich amplification threshold $F = f/2$ indicated. Extracted total resistance R, all three phases. Also in the bottom right, a prediction of where, for the low-resistance case, the system has for all circuits a negative resistance. For this prediction, the high resistance extracted total resistance R data for all three circuits is offset by the respective changes in R_{var}, and the maximum value of the three circuits is shown. The negative resistance lies along the resonance peak curve in Fig. 6 (main text). Note the two scales for positive and negative resistance.

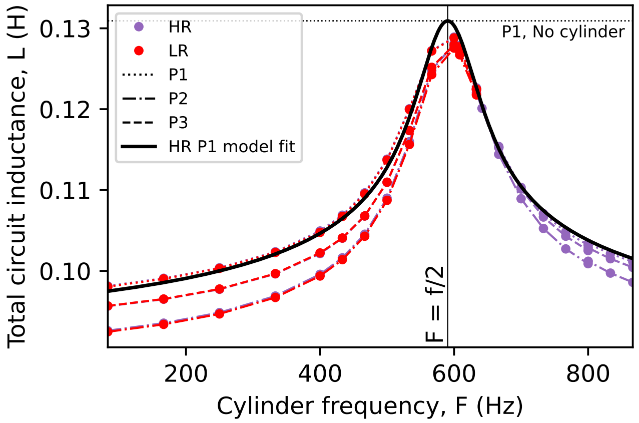

fig. S6. **Total inductance in the three circuits:** P1, P2, P3 at $f = 1181$ Hz for different cylinder rotation frequencies F. Purple dots are data for the high resistance measurements, and coinciding with these as expected, the red dots are the low resistance measurements. Various dashed lines are the linear interpolations between datapoints. The vertical line indicates the Zel'dovich threshold rotation, the dotted horizontal line the no-cylinder measured P1 value (0.131 H) in the high-resistance case. The Zel'dovich model fits to the P1 high resistance data are also plotted on top (black solid line), with the coupling strength $A = 0.397$ and a constant $L_0 = 0.131$ H being free fit parameters.

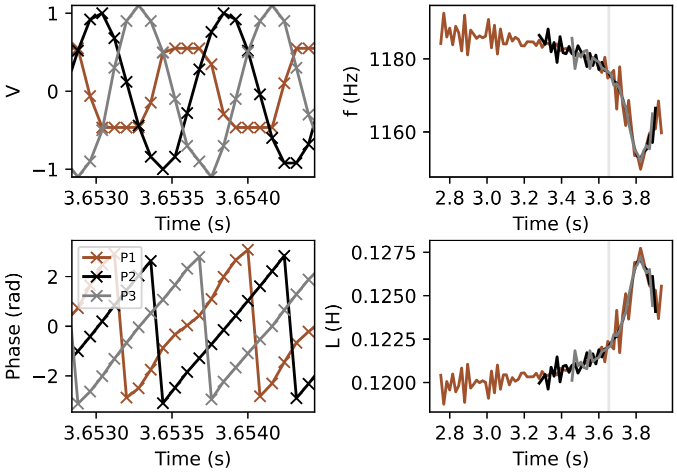

fig. S7. **Parameter extraction for** $F = +643$ **Hz dataset.** Signal, phase, instantaneous frequency, calculated inductance for the demand speed $F = +643$ Hz dataset shown in Fig. 4 (main text). The signal and phase are shown for a short time slice, the position of which is indicated by a vertical line in the f and L plots. The phase relationship between the circuits shows the excited EM mode has a definite rotation direction that is co-rotating with the cylinder.

Measured inductance

While the real part of the voltage measurements is used to calculate the resistance R in Fig. 3b of the main paper, the imaginary part can be used to calculate the inductance L, shown in fig. S6. Included is a fit of the inductance change in the P1 data to that predicted by the Zel'dovich theory. Here the same A value is used from the fit in Fig. 3b, and the L_0 offset is used as a free parameter, with the fitted value $L_0 = 0.131$ H matching the measured P1 no-cylinder value at that circuit frequency f, and the data following the model curve well.

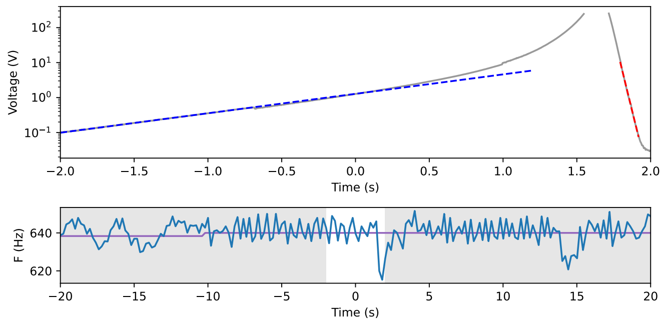

fig. S8. **Self-oscillation data for 640 Hz cylinder rotation.** Above shows the voltage measured over the coil. Here fits are done using Eq. 9 (Methods section) to regions where there is simple exponential behaviour to the amplification or the decay. Initial slope up (blue): 1.28 $e^{1.3t}$, slope down (red): (2.94e-03) $e^{-39.60(t-2)}$. Below is shown the recorded motor speed (blue), and the speed setting (purple). At around -10s the set speed is increased. The region with the white background corresponds to the time period of the voltage measurement above.

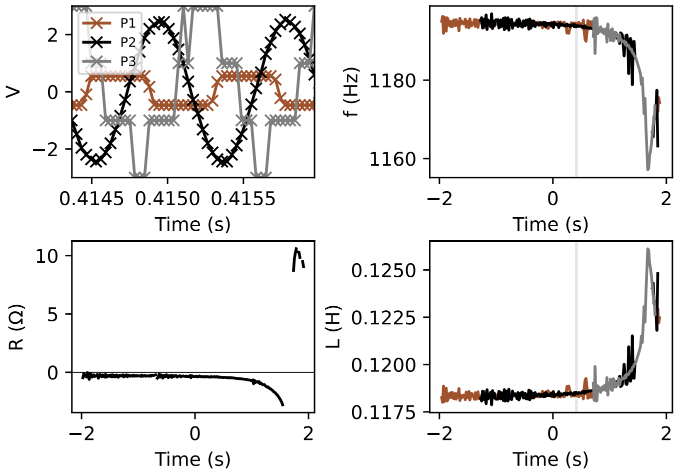

fig. S9. **Parameter extraction for $F = +640$ Hz dataset:** Voltage signal, instantaneous frequency, calculated resistance and calculated inductance for the demand speed $F = +640$ Hz dataset shown in fig. S8. The signal is shown for a short time slice, the position of which is indicated by a vertical line in the f and L plots.

Additional exponential amplification data

Fig. S7 shows the voltage signal, phase, instantaneous frequency and L values from the Fig. 4 (main text) F =+643 Hz driven data. Each circuit is measured with a different oscilloscope range to together track the exponentially increasing signal over more orders of magnitude. This is why for the time slice chosen, the voltage signal for P1 is saturated. The measured phases of the three circuits show when P1 has zero phase, P2 has -2.1 radians, P3 has +2.1 radians. This is the same 120° phase relationship between P1, P2 and P3 as in Table I used to create the co-rotating mode for a positive rotation speed. This shows the exponentially amplifying EM mode created by the instability has a definite rotation direction, and that it is co-rotating with the cylinder, as expected from the Zel'dovich model.

Fig. S8 shows additional exponential amplification data. In this measurement, the voltage is measured over the coils, rather than the 5Ω resistor. Due to the large impedance of the coils ($Z \approx \omega L$) this measurement saturates the ±300V max range of the oscilloscope in the peak super-exponential region, but allows more of the exponential region to be observed.

The speed data is also shown for this measurement. The speed data was taken while the resistors were on their low values, while the speed is stepped up slowly from below the instability threshold to just above it. The step in the purple line shows the moment of the step in speed prior to the observed signal. A few seconds later a signal emerges over the oscilloscope noise floor and is recorded in the time period -2 to +2s. As the signal peaks and saturates the

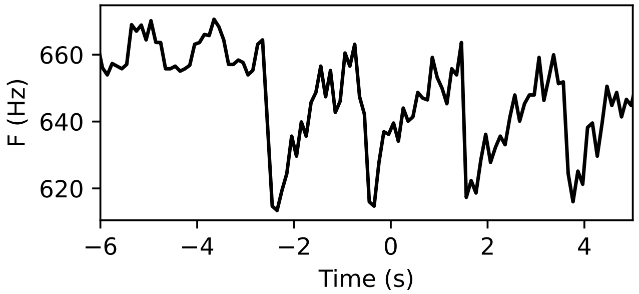

fig. S10. **Cylinder speed data:** Acquired from the motor corresponding to the voltage measurement shown in the inset of Fig. 4a. (main text).

drop in motor speed is recorded, as is the exponential decay of the signal. The motor speed picks up again, and a second drop in motor speed arises from the subsequent exponentially amplified signal peak. The signals are seeded from noise in the circuit lower than the oscilloscope noise on the measurement, at a minimum this is thermal noise, the thermal noise associated with the inductive coils would be $I_{\mathrm{r.m.s.}} = \sqrt{\frac{k_B T}{L}} \approx 1.8 \times 10^{-10}$ A (although this doesn't take into account the circuit resonance giving the noise a frequency dependence). In the experiment shown in Fig. S8 the speed is stepped up at -10.3 s; if the exponent fitted to the data was constant leading up to the data shown, the noise level at -10.3 s for the $\approx f = 1195$ Hz signal shown would have been $\approx 2 \times 10^{-9}$ A. In Fig. 4 of the main paper, back-extrapolating the main voltage signal shown would cross the thermal noise level at $t = -3.14$, which is just as the speed is reaching its target value. However, we can imagine at least other two mechanisms for the seed noise: (a) direct electromagnetic interference from environmental fields, such as those produced by power lines and harmonics; (b) vibrations of the stator coils, transduced via the earth field and Faraday effect. Note that in all cases, to have a finite effect, the seed noise must have components close to the resonance frequency of the stator circuit f.

Fig. S9 shows the corresponding signal, instantaneous frequency, extracted resistance and inductance. For this rotation speed just within the instability region the extracted negative resistance in the exponential region is very small, around -0.3 ohms, similar to that in Fig. 4d (main text).

To see how see how the speed of the cylinder affects the growth timescale of the amplification and the revival period of the peaks, we can compare the two measurements taken at F= +640 and +643 Hz with that taken at F=+660 Hz, the inset data of Fig. 4a (main text). There an initial cylinder speed of 660Hz (fig. S10) is obtained before R_{var} is switched to low values. The first excitation has an extracted negative resistance of -6.6 ohms in its constant-exponential region. After the first excitation drops the speed of the motor, the motor does not have time to fully get back up to the set original speed before the next growing excitation is above the oscilloscope noise floor (and not even before the self-halting mechanism kicks in). Therefore the subsequent peaks exponentially increase more slowly and so have a smaller extracted negative resistance of around -5 ohms (although as the speed is increasing in this case, there is no approximately constant exponent region).
